# Thyroid Sporadic Goiter with Adult Heterotopic Bone Formation

**DOI:** 10.1155/2015/806864

**Published:** 2015-12-01

**Authors:** Adriana Handra-Luca, Marie-Laure Dumuis-Gimenez, Mouna Bendib, Panagiotis Anagnostis

**Affiliations:** ^1^Service d'Anatomie Pathologique, APHP GHU Avicenne, UFR Médecine, Université Paris Nord Sorbonne Cité, 125 rue Stalingrad, 93009 Bobigny, France; ^2^Service Medecine Nucleaire, APHP GHU Avicenne, 93009 Bobigny, France; ^3^Division of Endocrinology, Police Medical Centre, Monastiriou 326, 54627 Thessaloniki, Greece

## Abstract

Thyroid heterotopic bone formation (HBF) in goiter is a rare finding. Five thyroid resection specimens were analyzed for HBF. The results were correlated with clinicomorphological features. All patients were women (33–82 years). The preoperative diagnosis was thyroid goiter or nodule. Treatment consisted in thyroidectomy and lobectomy (3 and 2, resp.). Microscopy showed sporadic nodular goiter. Malformative blood vessels and vascular calcifications were seen in intra- and extrathyroid location (5 and 3, resp.). The number and size of HBFs (total: 28) ranged between 1 and 23/thyroid gland (one bilateral) and 1 and 10 mm, respectively. Twelve HBFs were in contact with the thyroid capsule. Most were extranodular (21, versus 6 intranodular). The medical history was positive for dyslipidemia, hyperglycemia, renal dysfunction, and hyperuricemia (2, 3, and 3 cases and 1 case, resp.) without any parathyroid abnormality. In conclusion, thyroid HBF may be characterized by subcapsular or extranodular location, various size (usually ≥2 mm), and vascular calcifications and malformations. Features of metabolic syndrome and renal dysfunction may be present, but their exact role in the pathogenesis of HBFs remains to be elucidated.

## 1. Introduction

Heterotopic bone formation (HBF) is defined as extraskeletal bone formation. Thyroid HBF, frequently designated as bone metaplasia, occurs rarely in the thyroid, being reported both in goiter and in tumors such as adenomas and carcinosarcomas [1–10]. To our knowledge, seven cases of thyroid sporadic goiter with complete, adult HBF are reported in the English medical literature [2, 4, 7–10]. Here we report five additional cases of complete, adult HBF occurring in the context of sporadic thyroid nodular goiter.

## 2. Methods

Five thyroid resection specimens were analyzed for HBF as defined by the presence of lamellar bone trabeculae delimiting fat or fibrofat tissue with hematopoietic elements and capillaries. The number, size, and location of HBF foci (subcapsular or not, intranodular or not) were tabulated. Foci of ossification consisting only of bone trabeculae were considered separately. Thyroid parenchyma was also analyzed for nodules (hyperplastic, adenoma-type, or carcinoma), atrophy, necrosis, fibrosis, calcifications, inflammation, and vascular lesions (pseudoangioma lesions or vascular conglomerates, thrombosis, intima/media fibrosis and hyperplasia, and calcifications). Two thyroids were sampled quasi-entirely (Cases 1 and 2). Serial and/or multistep tissue sections were analyzed for the HBFs. The results were analyzed with regard to clinicomorphological features. The rank correlation Kendall test was used for evaluating the statistical significance of correlations (Medcalc v14, Belgium). A *P* value of less than 0.05 indicated statistical significance.

## 3. Results

The main features of the cases are demonstrated in Tables 1 and 2.

**Case 1 .**

**Figure 1:**
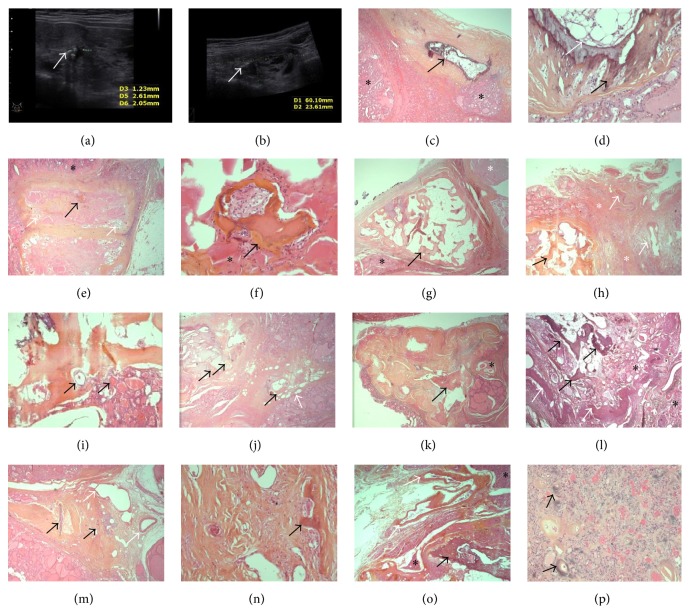
At ultrasound examination the thyroid showed several nodules and micro- and macrocalcifications (a, b: white arrows, Case 1). Microscopy showed in this case a HBF (heterotopic bone formation) focus in a thick rim of dense fibrosis (c, d: black arrow/HBF, asterisks/thyroid vesicles, and white arrow/intertrabecular fat with hematopoietic elements). Several HBFs were seen in Case 2 (e–k). A subcapsular nodule, largely fibrotic and atrophic, contained an infracentimetric HBF (e-f: black arrow/HBF, white arrow/nodular atrophic vesicles, and asterisk/reactive thyroid follicles). Another subcapsular HBF showed triangular shape and was situated in contact with an atrophic goiter nodule (g: black arrow/HBF, asterisk/thyroid vesicles, atrophic for some). A 3rd HBF was situated in contact with sheet-patterned fibrosis which contained large malformative vessels (h: black arrow/HBF, asterisks/thyroid vesicles, and white arrows/vessels). For this HBF, vesicles were at proximity and contact of bone trabeculae (i: black arrows). A 4th HBF was situated at proximity of intrathyroid adipose cells englobed in fibrosis (j: black arrow/HBF, white arrow/adipose cells). A 5th HBF was situated in a triangular-shaped zone of fibrosis, focally undulated, with an atrophic follicular nodule at contact (k: black arrow/HBF, asterisk/atrophic nodule). In Case 3 (l) a vaguely nodular zone, containing the HBF and thyroid vesicles, was delimited by undulated connective tissue (black arrows/HBF, asterisk/thyroid vesicles, and white arrows/undulated fibrosis with large vessels at contact). In Case 4 (m-n), the thyroid contained sheet-like fibrosis with large, malformative vessels at proximity and with ossification foci (m, n: black arrows/ossifications, white arrows/abnormal vessels). In Case 5 (o-p) the HBF was located in the subcapsular thyroid, at proximity to large malformative vessels (intra- and perithyroid) (o: black arrow/HBF, white arrow/malformative vessels, and asterisks/thyroid vesicles). The follicular nodule, situated at distance from the HBF, contained intervesicular disperse calcifications, some in the perivascular hyaline (p: black arrows).

The patient (51-year-old woman) had undergone a total thyroidectomy for toxic goiter. The patient was treated with carbimazole and thyroxine for 1.5 years. The medical history was positive for arterial hypertension and tachycardia as well as for cardiomegaly. There was no evidence of anemia. Foci of micro- and macrocalcifications were observed on thyroid ultrasound examination (Figure 1). Postsurgical hypocalcemia occurred and was treated with calcium supplementation. The patient was well at postsurgical consultation (after 3 months of follow-up).

Microscopy showed sporadic multinodular goiter with malformative, large, and tortuous blood vessels (intra- and extrathyroidal) intermingled with rare nerves. There were no vascular thromboses. Parenchymal nodules, several encapsulated, were hyperplastic and adenoma-like. Inflammation was moderate. Fibrosis was severe and extensive with a band-like pattern without extrathyroid extension. One HBF was identified (2.5 mm) with no ossification foci. Multifocal adipose involution was seen.

**Case 2 .**

The patient (33-year-old woman) had undergone a total thyroidectomy for goiter with trachea deviation. She was euthyroid. Smoking of 10 packs/year was noted. There was no evidence of anemia. Thyroid ultrasound examination showed foci of micro- and macrocalcifications (Figure 1). The patient was well at postsurgical consultation (after 0.5 months of follow-up).

Microscopy showed sporadic multinodular goiter with malformative, large, and tortuous blood vessels (intra- and extrathyroidal) intermingled with rare nerves. Vascular cavities with tuft-like projections were associated. There were no vascular thromboses. Parenchymal nodules, several encapsulated, were hyperplastic and adenoma-like. Several atrophic nodules, some with intranodular fibrocollagen, were also seen. Inflammation was moderate. Fibrosis was severe and extensive with a band-like pattern, containing or being at proximity of large blood vessels (intra- or extrathyroid), without extrathyroid extension. Twenty-three HBFs (2–10 mm) were identified with 11 ossification foci. Three extranodular HBFs were in direct contact with the capsule of fibroatrophic nodules. For two HBFs, band-like fibrosis connected malformative vessels to the HBF. On serial sections, two ossification foci revealed intertrabecular spaces and were thus diagnosed as HBFs. Multifocal adipose involution was seen as well as intrathyroid muscle tissue (the closest at 6.5 mm from the HBF).

**Case 3 .**

The patient (63-year-old woman) had undergone a thyroidectomy for goiter. The patient was euthyroid and was diagnosed with arterial hypertension and mitral stenosis. There was no evidence of anemia. Postsurgical hypocalcemia was treated with calcium. The patient was well at postsurgical consultation (after 1 month of follow-up).

Microscopy showed sporadic multinodular goiter with malformative, large, and tortuous blood vessels (intra- and extrathyroid) intermingled with rare nerves. There were no vascular thromboses. Parenchymal nodules, several encapsulated, were hyperplastic and adenoma-like. Calcifications of the internal elastic lamina and media (von Monckeberg sclerosis-type) were observed in the vessel wall, in peri- and intrathyroid locations [11]. Perivascular calcifications of calcipheresis-type were seen in hyperplastic nodules. Inflammation was mild as well as fibrosis. One HBF (10 mm) was identified. Abnormal blood vessels were seen around the HBF.

**Case 4 .**

The patient (83-year-old woman) had undergone left thyroidectomy for a cystic nodule with trachea deviation. The patient had been treated with carbimazole and thyroxine (15 days). The medical history was positive for arterial hypertension and osteoporosis. There was no evidence of anemia. The patient was well at postsurgical consultation (after 2 months of follow-up).

Microscopy showed sporadic multinodular goiter with malformative, large, and tortuous blood vessels (intra- and extrathyroidal) intermingled with rare nerves. Vascular cavities with tuft-like projections were associated. There were no thromboses. Parenchymal nodules, several encapsulated, were hyperplastic and adenoma-like. Calcifications of the internal elastic lamina and media (von Monckeberg sclerosis-type) were observed in the vessel wall. Perivascular calcifications of calcipheresis-type were also seen. Thyroid inflammation was moderate and fibrosis mild. Two HBFs were identified (1 and 9.5 mm) with 14 ossification foci. Abnormal blood vessels were seen around the largest HBF.

**Case 5 .**

The patient (71-year-old woman) had undergone right thyroidectomy for compressive cyst. The patient was euthyroid. The medical history was positive for arterial hypertension. The patient also showed vitamin D deficiency (10.1 ng/mL) as well as hyperuricemia and arthrosis and did not show anemia. An evacuatory punction was followed by reincrease in size of the nodule (3 months afterwards). Postsurgical hypocalcemia occurred and was treated with calcium supplementation. The patient was well at postsurgical consultation (after 3 weeks of follow-up).

Microscopy showed sporadic multinodular goiter with malformative, large, and tortuous blood vessels (intra- and extrathyroidal) intermingled with rare nerves. There were no vascular thromboses. Parenchymal nodules, several encapsulated, were hyperplastic and adenoma-like. Calcifications of the internal elastic lamina and media (von Monckeberg sclerosis-type) were observed in the vessel wall. Perivascular calcifications of calcipheresis-type were seen in hyperplastic nodules. There were no vascular thromboses. Inflammation was mild to moderate and fibrosis was mild. One HBF (8 mm) was identified. Abnormal blood vessels were seen around the HBF. Adipose involution was multifocal.

### 3.1. Heterotopic Bone Formation Foci Feature Analysis

The total number of HBF foci was 28. The number varied between 1 and 23 foci/thyroid specimen (bilateral: one) and size ranged from inframillimetric to 10 mm. Eighteen (64%) HBFs were ≥2 mm and six (21%) ≥5 mm. The shape varied: triangular (*n* = 2), oval (*n* = 7), or rounded (*n* = 19) with a trend for triangular HBF to correlate with increased size (*P* = 0.08, tau = 0.233). Twelve HBFs were subcapsular in the thyroid and six occurred in nodules (hyperplastic adenoma-like and one entirely fibroatrophic). When extranodular, HBFs were situated in or in contact with band-patterned fibrosis. Thyroid vesicles, atrophic or not, were in contact with three intranodular and five extranodular HBFs. The intertrabecular tissue was adipose or fibroadipose (seven and 21 HBFs, resp.), with osteoblast-rimming and megakaryocytes (in two HBFs each). Adipose involution foci were close to some HBFs in Case 2. Intranodular HBFs were more frequently ≥2 mm (4 versus 2 intranodular HBFs of <2 mm). Size correlated with subcapsular location (*P* = 0.02, tau = 0.308), presence of adipose intertrabecular spaces (as compared to fibroadipose spaces, *P* < 0.01, tau = 0.385), contact with thyroid vesicles (*P* = 0.01, tau = 0.320), and presence of adjacent dysmorphic/malformative vessels (*P* = 0.01, tau = 0.435).

## 4. Discussion

Here we report five cases of thyroid HBF occurring in the context of sporadic goiter in euthyroid or hyperthyroid patients. The diagnosis of such lesions was microscopic. The imaging diagnosis was difficult; both micro- and macrocalcifications occurred. Although the HBFs were frequently extranodular and more than 2 mm in size when intranodular, the imaging features do not allow the precise diagnosis of HBF-type lesions. Whether the subcapsular location, seen in approximately one-third of the HBFs, might be useful remains to be further studied. The main relevance of intrathyroid HBFs is morphological, microscopical. Unlike on ultrasound examination, a misdiagnosis of carcinoma may be made on frozen-section examinations due to the presence of osteoclast-like elements [12].

The histogenesis of such lesions remains a matter of debate. The various thyroid topography, intraparenchymal or subcapsular, of the HBF foci we have seen, occurring in sheet-like fibrosis, more frequently extranodular, suggests a nonneoplastic origin. The presence of multiple, bilateral foci, round to oval more frequently, suggests a dysmetabolic rather than an ectopic nature. The most plausible hypothesis is that of degenerative changes, similar to those reported in the femoral arteries and, less frequently in the carotid, at ages above 60 [13]. Although we have noted von Monckeberg sclerosis-type calcifications both in the media and in the internal elastic lamina in three thyroids, including in intrathyroid location, the morphological aspects we have seen do not indicate a direct vascular origin, as no direct transition zones from blood vessel calcifications to HBF foci were detected on the different serial and multistep tissue sections. Moreover, malformative vessels lacked within the HBFs and were rare at contact. However, von Monckeberg sclerosis-type calcifications were seen in a perithyroid large vessel at 5 mm from the HBF in Case 5. An abnormal blood perfusion in the context of enlarged, plunging, goiter-thyroids with modified thyroid-vessel reports, possibly resulting in hypoxia/ischemia might be a favoring factor, as suggested by the presence of sheet-like patterned fibrosis connecting the large malformative tortuous vessels with the HBF foci. The presence of several fibroatrophic vesicular nodules in contact with some HBFs was also highly suggestive of an ischemic nature. Clotting abnormalities were not detected, neither anemia nor hematological disease. Of interest would be the relatively increased frequency of reported cases with intrathyroid hematopoiesis (associated with myelofibrosis or anemia or not) as compared to that of thyroid bone metaplasia [10]. The extensive study of the quasi-totality of thyroidectomy specimens revealed numerous ossification foci (more than 10) in two of the cases, while hematopoietic elements without bone formation lacked. However no hematologic disease was detected in the cases we report. Dysmetabolic factors such as dyslipidemia, diabetes, or fluctuant hyperglycemia and hyperuricemia as well were diagnosed in our cases, younger or older, and could be incriminated in the histogenesis of HBF. Multifocal thyroid adipose involution was seen in three thyroids, the patients' body mass index being above 30. However these lesions were rarely in direct contact with the adipose intertrabecular spaces of the HBFs to explain a possible participation to the HBF genesis.

HBF in the context of abnormal parathyroid functioning has been reported recently in one case [8]. Although we have detected fibroadipose intertrabecular spaces and osteoblast-rimming in some HBFs, the patterns of these lesions were not specific neither for hyperthyroid bone formation and resorption nor for hyperparathyroidism-bone modelling [14]. We did not encounter parathyroid function abnormality and the parathyroids were normal preoperatively and during perioperative examination.

Interestingly, the intranodular perivascular or intervesicular pattern of some calcifications observed in some of the nodules suggests a relationship with renal dysfunction, at least for early/initial lesions. Thyroid inflammatory disease may be also incriminated although there was no significant inflammation at the time of surgery. Riedel thyroiditis was ruled out based on microscopic features of the lesions: fibrosis, although focally extensive in two thyroids, remained intrathyroid [15]. Extensive fibrotic scarring may follow the fine-needle aspiration procedure. This hypothesis was ruled out in the cases we report since the punctured nodule was at distance from the HBF. Postradiotherapy fibrosis may be incriminated in the HBF genesis in Case 4, the patient's breast carcinoma being treated with radiotherapy, however, nine years before the thyroid surgery. Other causative agents of extensive fibrosis, such as radioactive iodine treatment, were not identified in any of the cases. The rarity of thyroid HBF in thyroid goiters rules out also a possible abnormal iodine metabolism. In animals, bone abnormalities are reported to relate to a possible iodine uptake [16]. Interestingly, iodine deficiency can also result in growth abnormalities with destructive alterations in bone and bone marrow, with decrease in hydroxyproline, hexosamines, and phosphomonoesterase-I activities, as well as in disorders of phosphate-calcium metabolism [16]. Whether systemic relationships, possibly indirect, exist between the thyroid HBFs and systemic osteoarticular conditions (diagnosed in three of the cases) such as osteoporosis, chondrocalcinosis, dorsal or lumbar arthrosis, hyperuricemia, and vitamin D deficiency remains to be further investigated. Of note would be the fact that in experimental models on guinea pigs thyroid hormones may result in bone (without cartilage) formation when injected intramuscularly, by a possible osteoblast transportation in muscle fibroblasts [17]. This hypothesis requires further explorations, particularly in humans, despite the simplicity of our observations of thyroid vesicles in contact with HBFs as well as of intrathyroid muscle, however not in direct contact with the HBF.

In conclusion, HBF may occur in sporadic thyroid goiter. A subcapsular or extranodular location and size ≥2 mm may be useful for the imaging diagnosis. Histogenesis is multifactorial, dysmetabolic conditions, renal dysfunction or vascular abnormalities being possibly involved, without associated parathyroid pathologies. Whether a disturbed iodine metabolism can also be involved requires further investigation.

## Figures and Tables

**Table 1:** Clinical features of the 5 patients with adult bone metaplasia.

Case number	Age (years)	Gender	Euthyroid	Punction	Presurgical diagnosis	Cardiovascular disease	Dyslipidemia	Diabetes	Osteoarticular disease	Impaired renal function	BMI	Type of thyroid surgery	Morphological diagnosis	Postsurgical hypocalcemia
1	51	W	No	No	Toxic goiter	AHT, tachycardia cardiomegaly	NA	No	Odontoid chondrocalcinosis, C4–C7 arthrosis	No	31.3	Right and left thyroid lobectomies	Sporadic goiter	Yes

2	33	W	Yes	No	Multinodular goiter (trachea deviation)	No	NA	No	No	Yes	32	Total thyroidectomy	Sporadic goiter	No

3	63	W	Yes	No	Multinodular goiter	AHT, mitral stenosis	Yes	Yes	No	Yes	22.5	Total thyroidectomy	Sporadic goiter	Yes

4	83	W	No	No	Left cystic nodule (trachea deviation)	AHT	NA	No	Osteoporosis	Yes	26.4	Left thyroid lobectomy	Goiter with adenoma-like nodule	No

5	71	W	Yes	Yes^*∗*^	Compressive cyst	AHT	Yes	No	Arthrosis, serum vitamin D OH 25D1 D3 insufficiency, and hyperuricemia	No	41.9	Right thyroidectomy	Follicular adenoma, cystic change	Yes

BMI: body mass index, NA: nonavailable, W: woman, and AHT: arterial hypertension.

^*∗*^The punction was performed for evacuating the cyst (65 mL); no cytological analysis was performed (Case 5).

Hyperthyroidism was diagnosed in Cases 1 and 4 and treated by carbimazole and thyroxin for 1.5 years in Case 1 and by carbimazole only in Case 4 (for 15 days due to temporary drug unavailability).

Decreased serum creatinine was diagnosed in Case 2, hypocalcemia and hypoalbuminemia were diagnosed in Case 3, and renal failure was diagnosed in Case 4. The type of dyslipidemia was not available in Case 3 and consisted in hypercholesterolemia and hyper-LDL-emia in Case 5. Cases 4 and 5 showed fluctuant hyperglycemia. Case 3 diabetes was type II.

Case 4 showed a history of sigmoidectomy for diverticulosis (date NA), gastric resection for gastrointestinal stromal tumor (date NA), and breast cancer (treated by surgery, radiotherapy, and hormonotherapy). Case 5 showed a history of appendectomy and skin papillomas. Case 3 showed hypoacusia (prosthesis).

There was no alcohol abuse in any of the cases; smoking habits (10 PA) were noted in Case 2. A treatment with propranolol was known for Case 1 and with atorvastatin, metformin, Lectil, beta-histidine chlorhydrate, metformin, glimepiride, hydroxyzine (allergy to penicillin and cetirizine), alendronic acid, spironolactone, atenolol, and zolpidem for Case 4. Allergy to fish and amoxicillin was known in Case 5, to penicillin and cetirizine in Case 3, and to penicillin and aspirin in Case 4.

**Table 2:** Main morphological characteristics of the 5 thyroidectomy specimens.

Number	Thyroid weight (grams)	Thyroid volume (mm^3^)	Number of HBF foci (size, mm)	Number of ossification foci	Thyroid calcifications	Thyroid fibrosis	Thyroid inflammation	Vascular calcifications	Thyroid adipose involution
1	48	93.75	1 (2.5 mm)	0	1	Severe	Moderate	No	Multifocal

2	32	72	27 (2–10 mm)	11	1	Severe	Moderate	No	Multifocal

3	38	51	1 (10)	0	1^*∗*^	Mild	Mild	Intra-, perithyroid	No

4	115	195	2 (1 and 9.5 mm)	14	1^*∗*^	Mild	Moderate	Intra-, perithyroid	No

5	43	180	1 (8 mm)	0	1^*∗*^	Mild	Mild to moderate	Intra-, perithyroid	Multifocal

^*∗*^Cases 3, 4, and 5 showed also reticular and perivascular calcifications in hyperplastic nodules.

Normal parathyroid tissue was seen in the perithyroid adipose tissue in Case 1.
